# Does sodium bicarbonate infusion really have no effect on the incidence of acute kidney injury after cardiac surgery? A prospective observational trial

**DOI:** 10.1186/s13054-015-0906-9

**Published:** 2015-04-22

**Authors:** Anna J Wetz, Anselm Bräuer, Michael Quintel, Daniel Heise

**Affiliations:** Department of Anesthesiology, Emergency and Intensive Care Medicine, University of Goettingen, Robert-Koch-Str. 40, Goettingen, 37075 Germany

## Abstract

**Introduction:**

Postoperative acute kidney injury (AKI) is a frequently observed phenomenon after cardiac surgery with cardio-pulmonary bypass (CPB); this severe complication is associated with adverse patient outcomes. There are multiple mechanisms involved in AKI during cardiac surgery, including CPB-dependent hemolysis. An IV infusion of sodium bicarbonate, which leads to urine alkalization, may play a role in preventing AKI. Recently, several trials have investigated the effect of sodium bicarbonate and reported controversial results. The purpose of this investigation was to investigate the following question. Under what circumstances can sodium bicarbonate prevent postoperative AKI?

**Methods:**

We analyzed data from 342 patients undergoing CPB surgery at the University Hospital Goettingen, Germany. A total of 174 patients received a preemptive dose of sodium bicarbonate. Directly after the induction of anesthesia, the continuous infusion of 0.15 mmol/kg body weight/h was started and continued until 2 pm on the first postoperative day. Patients who were not treated with sodium bicarbonate formed the control group (n = 168). To verify the AKI risk configuration of each group, we surveyed risk factors and determined the commonly used clinical predictive score according to Thakar and colleagues. We recorded the concentration of free hemoglobin (fhb) to estimate the amount of CPB-dependent hemolysis. The definition of AKI was acquired by applying the AKI-network (AKIN) classification over the course of five postoperative days.

**Results:**

Patients who received the sodium bicarbonate infusion showed a significantly lower incidence (35.6 vs. 50%) of AKI than that of patients who did not receive the infusion (p = 0.01). AKIN levels 2 and 3 were also more frequent when sodium bicarbonate was not administered. Particularly, in the low-risk cohort (<3 *Thakar* points), the incidence of AKI was significantly reduced (26 vs. 46%) when patients received sodium bicarbonate (p = 0.01), whereas in the high-risk patients, no significant reduction was observed.

**Conclusion:**

In this study, we observed that low-risk patients particularly benefited from the preventive treatment with sodium bicarbonate. The incidence of AKI was significantly reduced in low-risk patients while there was no statistically significant difference in the high-risk patient cohort.

**Trial registration:**

DRKS00007616, Registered 12 December 2014.

## Introduction

Postoperative acute kidney injury (AKI) is a frequently observed phenomenon after cardiac surgery with cardiopulmonary bypass (CPB); this severe complication is associated with adverse patient outcomes and has extensive medical and economic consequences [1]. It is one of the most common organ dysfunctions encountered in intensive care medicine [2], and cardiac surgery is among its most frequent causes [3]. AKI requiring renal replacement therapy (RRT) has been reported in 5% of patients after cardiac surgery; 8 to 15% present increased serum creatinine of >1.0 mg/dl; and a discrete increase of creatinine of 25% relative to the baseline is found in 50% of patients [4]. Surgery with CPB increases the postoperative mortality by 2 to 8%; in the event of AKI, however, the mortality rate increases exponentially, reaching up to 60% for patients who require RRT [5,6].

There are multiple mechanisms involved in kidney injury during cardiac surgery, including ischemia reperfusion injury, perioperative hemodynamic instability, impaired renal blood flow, CPB-induced activation of inflammatory pathways, generation of reactive oxygen species and hemolysis [4,7]. Hemolysis during CPB is induced by the mechanical destruction of erythrocytes due to contact with the surfaces of the bypass circuit, high blood flow and pressure conditions, and cardiotomy suction [4,8]. Consequently, the released free hemoglobin (fhb) passes through the glomerulus after it exceeds the binding capacity of haptoglobin [9]. The fhb then causes tubular obstruction with met-hemoglobin casts and tubular cell necrosis. Furthermore, free iron is released, which is involved in the generation of reactive oxygen species [4].

The administration of sodium bicarbonate, which results in urinary alkalization, may thus help to prevent hemoglobin-associated pigment nephropathy. Urinary alkalization prevents tubular obstruction due to cast formation by reducing the conversion of hemoglobin to met-hemoglobin. It also reduces the endocytotic uptake of hemoglobin, thereby protecting against cell necrosis of the proximal tube. Furthermore, urinary alkalization protects from oxidant injury by shifting the equilibrium of the Haber-Weiss reaction towards a lower production of hydroxyl radicals and therefore limiting the production of further free radicals and other reactive species (for example, peroxynitrite and free-iron-mediated hydroxyl radical formation) [4,10]. Additionally, one might presume that in alkaline pH the physiological scavenging of free radicals is promoted; a direct scavenging ability of bicarbonate itself has also been suggested [11].

Recently, several trials and meta-analyses have investigated the clinical effects of sodium bicarbonate, yielding controversial results [7,12-18]. To investigate the circumstances under which sodium bicarbonate can prevent postoperative AKI in our patient cohort, we determined the influence of a perioperative intravenous (IV) infusion of sodium bicarbonate on the incidence of AKI after cardiac surgery.

## Methods

We analyzed data from 342 patients of the University Hospital Goettingen, Germany. Based on a pilot study [12] and a position paper of the Renal Failure working group of the collaborative group, Cardiothoracic Intensive Care of the German Society of Anesthesiology and Intensive Care Medicine (DGAI) and of the German Society of Thoracic and Cardiovascular Surgery (DGTHG) [19], a new standard operating procedure was implemented in our department that recommended a perioperative IV infusion of sodium bicarbonate. This change occurred while we carried out a prospective large observational study that recorded predictive factors for AKI after CPB and monitored the incidence of AKI over a five-day period following surgery (data not yet published). This observational study was approved by the local ethics committee of the University of Goettingen (number 21/6/09), and we used a subset of this study to analyze the influence of sodium bicarbonate.

All patients undergoing cardiac surgery with CPB were included in the study through their participation in the aforementioned observational clinical trial with informed patient consent. We excluded patients with incomplete datasets, patients who were younger than 18 years of age, patients with chronic renal failure requiring RRT before surgery, patients for whom cardiac surgery was planned to not include CPB, heart-transplant patients, and patients with preoperative extra-corporal membrane-oxygenation (ECMO).

To analyze the influence of sodium bicarbonate, we divided the study cohort into two groups. Group 2 (called the NaHCO_3_ group) received a preemptive dose of sodium bicarbonate in accordance with the aforementioned new standard operating procedure. While implementing the new standard administration of sodium bicarbonate at the end of the aforementioned observational trial, 225 patients, all of whom received sodium bicarbonate, were included in the observational study and formed the NaHCO_3_ group of this study. Directly after the induction of anesthesia, a continuous IV infusion of 0.15 mmol/kg body weight/h was started and continued until 2 pm on the first postoperative day, modified from the procedure reported in the pilot study of Haase and colleagues [12].

Group 1 was assigned as the control group and included the final 225 patients (in accordance with the size of group 2) who were enrolled in the observational study before the new standard operating procedure was implemented and who consequently did not receive sodium bicarbonate.

To verify the AKI risk configuration of both groups, we surveyed individual patients according to various risk factors and determined the commonly used clinical predictive score according to Thakar and colleagues [6], which demonstrated an acceptable ability to predict the risk of AKI [20,21]. The following biometrical characteristics, comorbidities and surgical factors were recorded: gender, age, left-ventricular ejection fraction (EF), congestive heart failure (CHF)/NYHA-classification, chronic obstructive pulmonary disease (COPD), insulin-dependent diabetes mellitus (IDDM), the use of a preoperative intra-aortic balloon pump (IABP), and the preoperative concentration of serum creatinine.

We documented the surgical procedures as coronary artery bypass grafting (CABG) only, valve surgery only, combined CABG-valve surgery, or other surgeries (for example, myectomy, aortic arch surgery, and CABG with maze procedures and CPB); primary surgery or repeat surgery, and elective or emergency surgery. We also documented the duration of CPB and the aortic cross-clamping (ACC) time as surgical factors. Additionally, we recorded the baseline concentration of fhb and the concentration of fhb after the arrest of CPB as the assumed nephrotoxic agent to estimate the level of CPB-dependent hemolysis.

Postoperatively, the definition and evolution of AKI was evaluated by applying the Acute Kidney Injury Network (AKIN) classification [22]. Daily AKIN levels were determined by the diuresis rate and the serum creatinine concentration. We considered the maximum AKIN level achieved by each patient during the observed time period from the day of surgery until the fifth postoperative day. Consequently, patients were categorized into groups without AKI (AKIN 0) and with AKI (AKIN 1 to 3). Additionally, the serum creatinine concentration on day 10 after surgery, the length of stay (LOS) in hospital, length of stay in an intensive care unit (LOS-ICU) and in-hospital mortality were recorded as outcome parameters. Further, fluid balances up to the first postoperative day were analyzed.

Risk stratification for AKI and the comparability between the NaHCO_3_ group and control group were accomplished and ensured via statistical analysis and included testing distributions with the Kolmogorov-Smirnov test and applying univariate analysis with the Mann-Whitney *U*-test and the chi-squared (chi^2^) test, as well as logistic regression. Because the fhb concentration (as a risk factor) was higher in the control group than in the NaHCO_3_ group, we performed a multivariate logistic regression analysis (using backward selection, entry criteria *P* <0.05, and exit criteria *P* >0.1) with the two factors fhb and sodium bicarbonate to verify the independent influence of sodium bicarbonate on the incidence of AKI. Receiver operating characteristic (ROC) analysis was used to determine a cutoff point for the Thakar score to discriminate between patients at low risk and high risk of developing AKI (maximizing the sensitivity and specificity using the Youden index).

To analyze the effect of sodium bicarbonate, the incidence of AKI (according to the AKIN classification) was compared between the control group and the NaHCO_3_ group using the chi^2^ test. We also analyzed the effect of sodium bicarbonate in subgroups, categorized by the surgery procedure and low or high AKI risk (Thakar score). Non-normally distributed data were expressed as median and IQR, and normally distributed data as mean and standard deviation. A *P*-value <0.05 was considered statistically significant. Statistical analyses were carried out using Statistica (Version 10, 1984–2011, StatSoft Inc., Tulsa, OK, USA) and MedCalc (Version 12.4.0.0. for Windows XP/VISTA/7/8, MedCalc Software, Ostend, Belgium).

## Results

After exclusions due to incomplete data, 342 patients remained in the analysis. The study population consisted of 74% male patients with a median age of 70 years. They mostly underwent CABG surgery, followed by valve surgery, combined valve and CABG surgery and other types of surgery with CPB. Emergency or previous surgeries were performed in 3% of patients. The incidence of the preoperative risk factors EF <35%, COPD and IDDM were 10, 14 and 16%, respectively. CHF was recorded in more than 40% of the patients. Preoperative IABP was used in one patient. The median serum creatinine was 0.96 mg/dl, whereas serum creatinine >1.2 mg/dl was recorded in 23% of the patients. Individual predictive score points according to Thakar *et al*. ranged from 0 to 10 points [6], with a median score of 2 points, and 18% of the patients presented with 0 Thakar points (and were accordingly assigned as very low risk for AKI requiring RRT). The median CPB time was 115 minutes, and the median ACC time was 73 minutes. The baseline concentration of fhb was 9 mg/dl, which increased to a median of 50 mg/dl after ending CPB (Tables 1 and 2).

**Table 1:** **Descriptive statistics of the study cohort and results of the univariate analysis for each risk factor**

	**All**		**Control group**		**NaHCO**_**3**_ **group**		
	**Number**	**%**	**Number**	**%**	**Number**	**%**	***P*** **-value**
Biometric data							
Female	90	26.32	43	25.6	47	27.01	0.86
Male	252	73.68	125	74.4	127	72.99	0.86
Comorbidities							
IABP	1	0.29	0	0	1	0.57	0.98
Ejection fraction <35%	35	10.23	20	11.9	15	8.62	0.41
IDDM	49	14.33	23	13.69	26	14.94	0.86
COPD	56	16.37	25	14.88	31	17.82	0.56
Creatinine >1.2 mg/dl	79	23.1	40	23.81	39	22.41	0.86
Congestive heart failure	145	42.4	68	40.48	77	44.25	0.55
Surgery characteristics							
CABG	182	53.22	83	49.40	99	56.90	0.27
Valve	53	15.5	31	18.45	22	12.64	0.27
CABG + valve	40	11.7	23	13.69	17	9.77	0.43
Other surgery	67	19.59	31	18.45	36	20.69	0.63
Repeat surgery	9	2.63	2	1.19	7	4.02	0.19
Emergency surgery	9	2.63	5	2.98	4	2.30	0.96

Numbers and percentages for biometric data, comorbidities, and surgery characteristics are presented. Statistical analysis was performed using the chi^2^ test. IABP, intra-aortic balloon pump; IDDM, insulin-dependent diabetes mellitus; COPD, chronic obstructive pulmonary disease; CABG, coronary artery bypass graft.

**Table 2:** **Distributions of non-categorical data**

	**Control group**			**NaHCO**_**3**_ **group**			
	**Median**	**IQR**	**Range**	**Median**	**IQR**	**Range**	***P*** **-value**
Age, years	70	62, 74	34 to 84	70	60, 74	42 to 88	0.64
Creatinine, mg/dl	0.96	0.81, 1.18	0.5 to 2.1	0.97	0.81, 1.13	0.54 to 4.57	0.26
Score points	2	1.0, 3.5	0 to 10	2	1.0, 4.0	0 to 10	0.86
ACC time, minutes	74.5	61.5, 97.5	17 to 214	69.5	52, 91	29 to210	**0.03**
CPB time, minutes	116	94, 147.5	42 to 310	112.5	89, 138	45 to 313	0.06
fhb at baseline, mg/dl	9	8.0, 11.0	4.0 to 93	9	8.0, 11.0	5.0 to 55	0.18
fhb after CPB, mg/dl	55	40,73	7.0 to 187	45	34, 62	11 to 130	**0.001**

Distributions of non-categorical data, which are expressed as median, IQR, and range. Tests of the distributions of age, cardiopulmonary bypass (CPB) time, concentration of serum creatinine, and concentration of free hemoglobin (fhb) at baseline and at the end of CPB were performed by logistic regression. The Mann-Whitney *U*-test was used to evaluate the distribution of score points. The fhb concentration after CPB was significantly lower in the NaHCO_3_ group, and the aortic cross-clamping (ACC) time was significantly shorter.

Group 1 consisted of 168 patients who did not receive sodium bicarbonate therapy, and group 2 consisted of 174 patients who received an IV infusion of sodium bicarbonate.

Statistical analysis of each risk factor had the following distributions in the control and NaHCO_3_ groups: univariate analysis of gender, age, COPD, IDDM, EF <35%, CHF and IABP revealed no significant differences in distributions between the cohort of patients who received sodium bicarbonate and the cohort who did not (*P*-values are also presented in Table 1). Neither were significant differences in distributions observed in univariate analyses of preoperative serum creatinine concentration, surgery type, emergency or repeat surgery or CPB time. Individual score points were also equally distributed. However, patients in the control group were found to have a higher median concentration of fhb (55 versus 45 mg/dl) and a five-minute longer ACC time than that of patients who received sodium bicarbonate treatment (Tables 1 and 2).

**Figure 1:**
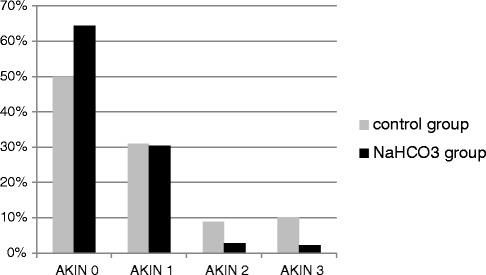
Acute Kidney Injury Network (AKIN) levels.

AKI was identified in 42.7% of the 342 patients (Table 3). In the group who received preemptive treatment with sodium bicarbonate, the AKI incidence was 35.6%, compared to 50% in the group without treatment, indicating that patients who received sodium bicarbonate showed a significantly reduced incidence of AKI compared with that of patients who were not administered sodium bicarbonate (*P* = 0.01). In particular, AKIN levels 2 and 3 were identified more frequently in the group without sodium bicarbonate treatment, whereas the incidence of AKIN level 1 remained unchanged (Figure 1). We found that 62% of the patients classified as AKIN 1 still had serum creatinine concentrations >1.2 mg/dl on day 10 after surgery. However, there was no significant difference between patients with or without sodium bicarbonate treatment (*P* = 0.840). We also found no significant difference in the quotient of the day-10 value to the baseline concentration between both groups (*P* = 0.370).

**Table 3:** **Acute Kidney Injury Network (AKIN) classification in the total cohort (342 patients), in the control group and in the NaHCO**_**3**_ **group**

	**All**		**Control group**		**NaHCO**_**3**_ **group**	
	**Number**	**%**	**Number**	**%**	**Number**	**%**
AKIN 0	196	57.31	84	50.00	112	64.37
AKIN >0	146	42.69	84	50.00	62	35.63
AKIN 1	105	30.70	52	30.95	53	30.46
AKIN 2	20	5.85	15	8.93	5	2.87
AKIN 3	21	6.14	17	10.12	4	2.30

Comparing the in-hospital mortality rate and LOS (for both hospitalization and intensive care) revealed no significant differences between patients who received sodium bicarbonate and those without treatment. The median LOS was 11 days, and the mortality rate was 2.6% (Table 4).

**Table 4:** **Length of hospital stay in days, length of intensive care stay, and in-hospital mortality in the control group and in the NaHCO**_**3**_ **group**

	**Control group**	**NaHCO**_**3**_ **group**	***P*** **-value**
Length of stay, days, median (IQR)	11 (8, 15)	11 (8, 15)	0.7834
Length of stay in ICU, days, median (IQR)	2 (1, 4)	1 (1, 4)	0.1969
Mortality, number (%)	3 (1.79)	6 (3.45)	0.5050

The Mann-Whitney *U*-test and chi^2^ tests revealed no significant differences between the two groups.

The mean fluid balance up to the first postoperative day was significantly higher in the group of patients who received sodium bicarbonate than in the control group (2806 ± 1607 ml versus 1517 ± 1622 ml, *P* <0.0001). However, there was no difference between patients with and without AKI (*P* = 0.244) or high-risk and low-risk patients (*P* = 0.319).

Multivariate analysis of the two factors (sodium bicarbonate and fhb after finishing CPB) demonstrated that receiving sodium bicarbonate has not only a significant but also an independent effect on the prevention of AKI. The absence of sodium bicarbonate contributed to AKI, with an odds ratio of 1.57 (95% CI 1.0009, 4.4494) (Table 5).

**Table 5:** **Multivariate analysis**

	***P*** **-value**	**Odds ratio**	**95% CI**
Sodium bicarbonate	0.0496	1.5657	1.0009, 4.4494
Free hemoglobin	0.0001	1.0183	1.0089, 1.0277

Influence of the absence of sodium bicarbonate treatment and free hemoglobin at the end of cardiopulmonary bypass on acute kidney injury.

**Figure 2:**
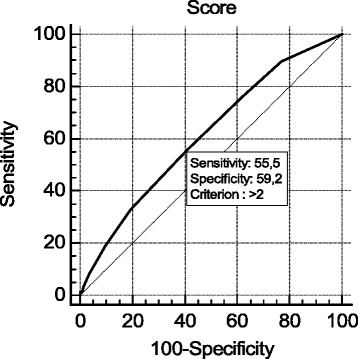
Receiver operating characteristic analysis.

To analyze the effect of sodium bicarbonate in low-risk and high-risk AKI patients, we performed a ROC analysis to subdivide the cohort. The cutoff point of the Thakar score was set at 3 points to discriminate between the low-risk and high-risk patients (Figure 2). Accordingly, we divided the cohort in two sub-cohorts: 161 patients with a score ≥3 points were assigned as high-risk patients, and patients with scores <3 points were defined as low-risk patients (n = 181). In the low-risk cohort, the AKI incidence was significantly lower (26 versus 46%) when patients received sodium bicarbonate; not receiving the treatment contributed to AKI with an OR of 2.34 (95% CI 1.25, 4.36). In the high-risk cohort, the AKI incidence was lower in the NaHCO_3_ group than in the control group (46 versus 55%), but the difference was not significant (Table 6).

**Table 6:** **AKI incidence in the control group and the NaHCO**_**3**_ **group in low-risk and high-risk AKI patient**

**Low-risk (score points 0 to 2)**	**Control group (n = 90)**		**NaHCO**_**3**_ **group (n = 91)**		
	**Number**	**%**	**Number**	**%**	***P*** **-value**
AKIN 0	49	54.44	67	73.63	**0.0113**
AKIN >0	41	45.56	24	26.37	
**High-risk (score points 3 to 10)**	**Control group (n = 78)**		**NaHCO**_**3**_ **group (n = 83)**		
	**Number**	**%**	**Number**	**%**	***P*** **-value**
AKIN 0	35	44.87	45	54.22	0.3042
AKIN >0	43	55.13	38	45.78	

The acute kidney injury (AKI) incidence was significantly lower in the low-risk cohort when patients received sodium bicarbonate.

After categorizing the patient cohort by surgical procedure, we found that the AKI incidence in patients undergoing CABG surgery was reduced significantly (52 versus 34%) when sodium bicarbonate was administered. We also found that patients who underwent CABG surgery had a lower median concentration of fhb compared to patients who underwent other types of surgery. For the other surgical procedures, no significant decrease in the incidence of AKI was detected (Table 7).

**Table 7:** **Incidence of AKI in the control group and the NaHCO**_**3**_ **group according to the surgical procedure**

**Surgery type**	**AKIN**	**Control group**		**NaHCO**_**3**_ **group**			**Free hemoglobin**	
		**Number**	**%**	**Number**	**%**	***P*** **-value**	**Median**	**IQR**
CABG	AKIN 0	40	48.2	65	65.7	**0.0261**	43.5	32, 62
	AKIN >0	43	51.8	34	34.3			
Valve	AKIN 0	18	58.1	15	68.2	0.6447	54	37.25, 66.25
	AKIN >0	13	41.9	7	31.8			
CABG + valve	AKIN 0	10	43.5	9	52.9	0.7885	60	49.5, 78.5
	AKIN >0	13	56.5	8	47.1			
Other	AKIN 0	16	51.6	23	63.9	0.4428	57	42, 78
	AKIN >0	15	48.4	13	36.1			

In the subgroup of coronary artery bypass graft (CABG) surgery patients, the acute kidney injury (AKI) incidence was reduced significantly when patients were treated with sodium bicarbonate. The median concentrations and IQRs of free hemoglobin for each subgroup are also presented. AKIN, Acute Kidney Injury Network classification.

## Discussion

In this study, we analyzed the influence of perioperative treatment with sodium bicarbonate IV on the incidence of AKI after CPB. We observed that the incidence of AKI was significantly lower in the group of patients who received the sodium bicarbonate treatment: 50% incidence of AKI (control group) versus 35.6% (NaHCO_3_ group). Apart from the fhb concentration and ACC time (which can possibly be attributed to the slightly increased number of combined CABG-valve operations in the control group), none of the comorbidities, or biometric or surgical factors were significantly different between the two groups. Although the fhb concentration was slightly higher in the control group, an independent, significant influence of sodium bicarbonate on AKI was demonstrated. Accordingly, our results showed that the IV infusion of sodium bicarbonate significantly reduced the postoperative AKI incidence after surgery with CPB in our study population, especially in patients with a lower preoperative risk of renal failure.

Preventive and therapeutic effects of sodium bicarbonate have been under discussion for a long time, dating back to the 1980s [23]. In the past, several studies have been published about the preventive use of sodium bicarbonate in the context of contrast-induced nephropathy [24]; however, in 2009, Haase and colleagues were the first to publish a randomized, double-blind, placebo-controlled trial examining cardiac surgery with CPB [12]. Potential side effects of sodium bicarbonate include increased plasma sodium concentrations and metabolic alkalosis [12,14]. In the present study, patients who received sodium bicarbonate had a significantly higher pH level of 7.41 (IQR 7.38, 7.44), in contrast to 7.39 (IQR 7.36, 7.42) in the control group, on the day of surgery and the first postoperative day (*P* <0.0001). No patient developed a plasma sodium concentration >150 mmol/l; marginal hypernatremia (>145 < 147 mmol/l) occurred in two patients.

Our results consequently support the hypothesis of Haase *et al*. 2009, indicating that sodium bicarbonate is an effective, simple, practical, and inexpensive therapeutic measure to prevent the nephrotoxic effects of CPB-dependent hemolysis [4,12,25]. However, other published trials have failed to reconfirm the results of the pilot study. None of those trials except one found that a sodium bicarbonate infusion reduced AKI incidence after cardiac surgery with CPB [7,12-18].

Based on the findings of the pilot study, the administration of sodium bicarbonate was implemented in our department of the University of Goettingen, Germany. We evaluated the effect of sodium bicarbonate infusion without randomization while the new standard operating procedure was being established. After the implementation of this new standard operating procedure, all patients received sodium bicarbonate and therefore formed the NaHCO_3_ group. As described above, the control group included patients who underwent cardiac surgery prior to the implementation of the standard operating procedure and who therefore did not receive sodium bicarbonate. The data in the present study originated from a large observational prospective study to evaluate AKI risk after cardiac surgery, which represents high-quality data recruitment. Despite the limitation that the treatment was not randomized, this study analyzed the effects of sodium bicarbonate on a large sample size of nearly 350 patients.

AKI was determined over five postoperative days using the AKIN classification, which is an objective, comparable, and precise definition of AKI. In contrast to other studies, we did not further apply AKI criteria nor record the use of diuretic therapy. In our department, we pursue a strategy that limits the use of diuretics. During surgery, during the first 24 hours after surgery and during the manifestation of oliguria to anuria, we refrained from administering any diuretics because several studies, including prospective randomized trials, have shown a worsening of renal function due to early diuretic use in cardiac surgical patients [26-28]. Even if diuretics were administered, it can be assumed that they had no effect on serum creatinine, allowing an accurate reflection of the extent of AKI even under diuretic therapy. Because the worse of the two criteria (urinary output and serum creatinine) is applied to identify the AKIN stage (as in our trial), diuretics should not have an effect on the AKIN classification.

The mean fluid balance up to the first postoperative day was significantly higher in the group of patients who received sodium bicarbonate than in the control group. However, there were no differences in fluid balances between patients with and without AKI or high-risk and low-risk patients. It is possible that dilution effects may have influenced the results. A lower incidence of AKIN 1 in the sodium bicarbonate group, consistent with lower serum creatinine concentrations, might partly be explained by a dilution effect due to higher fluid balances. Second, higher fluid balances may have directly contributed to the prevention of AKI. However, as there were no differences in fluid balances between patients with and without AKI, and high-risk and low-risk patients, respectively, our results still indicate a direct preventive effect of sodium bicarbonate on AKI.

The administration of sodium bicarbonate, leading to urinary alkalization, is assumed to prevent hemoglobin-associated pigment nephropathy. Because fhb is part of the pathophysiological pathway of pigment nephropathy [4], we found it predominantly important to determine the intraoperative concentration of fhb. Because none of the previously published trials determined the fhb concentration, our study presents a new and important perspective by analyzing fhb concentrations, assuming this to reflect nephrotoxicity in pigment nephropathy. Due to CPB-induced hemolysis, released fhb causes tubular obstruction and tubular cell necrosis. Urinary alkalization by sodium bicarbonate thus prevents tubular obstruction by reducing the conversion of hemoglobin to met-hemoglobin and reducing the endocytotic uptake of hemoglobin, hence protecting against tubular cell necrosis. Urinary alkalization protects against oxidant injury by limiting the production of free radicals and other reactive species by a pH-dependent shifting of the equilibrium of the Haber-Weiss reaction [4,10]. One might presume that in an alkaline milieu, the physiological scavenging processes of free radicals are promoted; a direct scavenging ability of bicarbonate itself has also been suggested [11]. Observing the concentration of fhb revealed a median of 50 mg/dl after ending CPB, which might represent an optimal interventional frame. Comparing this concentration range to values reported in the literature shows that (extremely) different conditions have been reported, ranging from 65 mg/dl [29]; 54.9 ± 27.1 mg/dl; and 46 ± 25.4 mg/dl after open-heart CPB surgery (depending on the use of various suction devices) [30] to 104.4 ± 36.5 mg/dl (patients without AKI) and 289.0 ± 37.8 mg/dl (patients with AKI) [31] and 120 ± 73 mg/dl during CPB [32]. We observed that sodium bicarbonate decreased the incidence of AKI in patients with moderate concentrations of fhb (for example, patients subjected to CABG surgery only), which implies that sodium bicarbonate may only protect against pigment nephropathy to a certain extent. Once fhb rises to high levels (for example, after a long CPB time, due to combined valve and CABG surgery), it might exceed the degree of possible protection afforded by sodium bicarbonate. In this regard, other causes of AKI may predominate over the role of fhb and thereby keep the role of sodium bicarbonate less efficient.

Because fhb was not observed and analyzed in previously published studies, it is possible that due to various surgery conditions the fhb concentrations were considerably higher and thus, not within a frame indicating that treatment with sodium bicarbonate would still be effective. Considerably more patients from the trials of Heringlake, McGuiness and Haase *et al*. underwent valve surgery (30, 51 and 48%) with assumed higher concentrations of fhb than were observed in our patient population. In contrast, we included only 27% of the patients with valve surgery (valve only and combined with CABG) but more than 50% of the patients with CABG-only surgery (versus 38, 29 and 18%, respectively) [13,15,16]. Moreover, we showed that particularly for patients undergoing CABG surgery, having a lower median concentration of fhb was associated with experiencing a greater benefit from treatment with sodium bicarbonate, a finding which was also just recently shown in a meta-analysis by Bailey and colleagues [7].

We found a slightly higher median concentration of fhb in the control group (possibly assuming more harm due to fhb) and therefore performed a multivariate analysis with the two factors sodium bicarbonate and fhb, which demonstrated that the administration of sodium bicarbonate had an independent, significant preventive influence on AKI. Consequently, this result highlights our finding that sodium bicarbonate reduces the incidence of AKI.

Comparing patient outcomes with regard to in-hospital mortality rates and LOS (hospitalization and intensive care) revealed no significant differences between patients who received sodium bicarbonate and those who did not, although the mortality rate was higher in the NaHCO_3_ group. Therefore, we cannot support the findings of Haase *et al*. [16], who reported significantly increased mortality in the NaHCO_3_ group. The other reported trials also did not find differences in the mortality rate (0 to 4%), supporting our result of 3% [12-15]. Indeed, in one trial, a mortality rate of 0% was found in the NaHCO_3_ group in contrast to 4% in the control group [14]. A significantly prolonged duration of treatment in intermediate-care and high-dependency units in the sodium bicarbonate group was observed by Heringlake and colleagues [13], but this finding was not reported elsewhere [12,14-16].

Considering the positive preventive effect of sodium bicarbonate, the question arises whether sodium bicarbonate also has an effect on the development of chronic kidney disease. Therefore, we analyzed the serum creatinine concentration on day 10 after surgery, as one parameter indicating persisting or chronic kidney disease. Contrary to our hypothesis, there were no significant differences between patients classified as AKIN 1, with or without sodium bicarbonate treatment. Thus, in our rather small patient cohort we could not demonstrate a preventive effect for sodium bicarbonate on the development of chronic kidney disease; future larger trials may analyze this outcome parameter.

Another difference between the studies can be found in the administration of sodium bicarbonate; we did not apply an additional bolus before starting the maintenance dose as in three other trials [12,13,15]. However, the sodium bicarbonate infusion was started directly after the induction of anesthesia before beginning surgery, and the patients received a continuous infusion of sodium bicarbonate until 2 pm on the following day. In two trials, the infusion was also applied at a constant rate, beginning with the induction of anesthesia and continuing for 24 hours, or beginning 1 hour preoperatively and continuing for only 6 hours after CPB, respectively [14,16]. The fact that we applied sodium bicarbonate over a longer time period may have improved the postoperative conditions, and a bolus-maintenance application may not be the optimal approach.

We found an AKI incidence close to 45%, although we not only focused on high-risk patients but also included all patients with CPB surgery. This choice resulted in the absence of risk factors for AKI (according to Thakar *et al*. [6]) in 18% of the study cohort. Analyzing the effects of sodium bicarbonate in the low-risk and high-risk patient cohorts (according to the Thakar score) revealed that in particular, low-risk patients with fewer than three score points benefitted from treatment with sodium bicarbonate. In the low-risk cohort, the AKI incidence was only 26% in the NaHCO_3_ group versus 46% in the control group (*P* = 0.01). This distinction may explain why trials that only included patients at high risk for AKI have failed to demonstrate any benefit of sodium bicarbonate therapy [14-16].

Bailey and colleagues suggest that, in contrast to the low-risk patients, AKI in high-risk patients is not primarily related to CPB time but is closely related to hemodynamic perturbation [7], indicating a reduced therapeutic benefit for sodium bicarbonate. Because the development of CPB-associated AKI is multifactorial, AKI worsens in tandem with increasing numbers of risk factors. Thus, the prevention of pigment nephropathy through the use of sodium bicarbonate, which addresses only one mechanism of AKI, becomes less effective. Our findings led to the conclusion that sodium bicarbonate can be a preventive measure, especially in low-risk AKI patients and patients undergoing CABG surgery, whereas it may not avert AKI in high-risk patients.

## Conclusion

In this study, we observed that low-risk patients particularly benefited from preventive treatment with sodium bicarbonate. In the low-risk cohort (with <3 Thakar points) and in CABG patients, the AKI incidence was significantly lower when patients received sodium bicarbonate, whereas in high-risk patients, the effect on the incidence was not significant.

## Key messages

- Patients with sodium bicarbonate IV infusion (n = 174) had significantly lower incidence of AKI than patients without treatment (n = 168): 35.6 versus 50.0%, *P* = 0.01.
- In the low-risk cohort (with <3 Thakar points; n = 181), the AKI incidence was significantly lower (26 versus 46%) when patients received sodium bicarbonate (*P* = 0.0113).
- In the high-risk cohort (n = 161), the AKI incidence was lower in the NaHCO_3_ group than in the control group (46 versus 55%), but the difference was not significant (*P* = 0.3042).
- The AKI incidence in patients undergoing CABG surgery was significantly lower in the NaHCO_3_ group (*P* = 0.0261). For other types of surgery, the AKI incidence was decreased, but not significantly.
- The baseline concentration of fhb was 9 mg/dl, which increased to a median of 50 mg/dl after ending CPB.

## Abbreviations

ACC: aortic cross-clamping
AKI: acute kidney injury
AKIN: acute kidney injury network (classification)
CABG: coronary artery bypass grafting
CHF: congestive heart failure
COPD: chronic obstructive pulmonary disease
CPB: cardiopulmonary bypass
ECMO: Extracorporal membrane-oxygenation
EF: ejection fraction
fhb: free hemoglobin
IABP: intra-aortic balloon pump
IDDM: insulin-dependent diabetes mellitus
IV: intravenous
LOS: length of stay
LOS-ICU: length of stay in the intensive care unit
NaHCO_3_: sodium bicarbonate (group)
ROC: receiver operating characteristic
RRT: renal replacement therapy
